# A Practical Treatise on Abdominal Hernia

**Published:** 1846-04

**Authors:** 


I. A Practical Treatise on Abdominal Hernia. By *Thomas Pridgin Teale*, F.L.S., Surgeon to the Leeds General Infirmary. With numerous Illustrations. Octavo, pp. 380. London, 1846.

II. A System of Surgery. By *J. M. Chelius.* Translated by *John F. South.* Part IX. Ruptures. 1846.

We must dissent from Mr. Teale's opinion, that an opening exists for a new work on hernia, bearing the character of a text-book for students and of reference for practitioners. Any elucidation of the few debateable points in the nature and management of this affection from the pen of a practical surgeon will at all times be acceptable; but another general work upon the subject was certainly not requisite for those having access to the books of Lawrence and Astley Cooper, and the Surgical Dictionary of Samuel Cooper. It seems, however, that the author originally prepared his work for the Cyclopædia of Surgery, of which it would have formed an appropriate portion; and, as that publication (to the regret of those who perused or purchased its early numbers) is defunct, he has brought it out in a separate form. It may be thought rather the author's and publisher's than the reviewer's business to decide whether a sound discretion has been used in issuing it as a substantive work: but in this opinion we can scarcely agree, as we consider our function consists not only in indicating what is good, valuable, or the contrary, in new works, but also in informing our readers how far such information has superseded that contained in the books they already possess. The sale of medical works of original research is already far too limited to allow of the indiscriminate recommendation of such as approach the character of compilations on the score of mere repetition of information, however important and essential. Supposing a new text-book on Hernia had been a *desideratum*, we are free to admit Mr. Teale has produced a very good one. Copiousness of detail does not prevent succinctness and clearness of description, the various difficulties of diagnosis are carefully appreciated, and the different means of relief judiciously set forth.

We are pleased to find that South (we follow his own invariable rule of omitting the ordinary prefix) has hitherto avoided the want of punctuality, so common now-a-days with authors of books published in parts, and so annoying to their readers, and we hope the work will meet with a commensurate sale. It deserves it on account of the industry and ability with which it is composed; although, when we see the large proportion of the notes compared to the text, we cannot but again regret that the translator did not write an original work, in the place of patching up the somewhat imperfect original.

We will advert to the opinions of these authors upon one or two of the most important points; and first, upon—

1. *The Operation without opening the Sac.*—Although this was performed by Petit so long ago as 1718, and has been practised by Monro and Cooper in large herniæ, yet it is to Key that we are indebted for placing it in its present condition in professional estimation. Indeed, according to Mr. South, Sir A. Cooper was not in the habit of performing this operation during the latter period of his career, nor does he recommend it in his Lectures as he did in the early editions of his work on Hernia. Key's recommendation of it is founded chiefly upon the mischief he believes arises from the exposure of the inflamed and strangulated bowel to the air and light, and to handling. When peritonitis ensues upon an operation, it is found to spread from the strangulated portion of the bowel to the rest of the peritoneum, and not from the incisions of the sac. Lawrence, however, believes the peritonitis is induced, not by exposure of the intestine, but by the pressure made by the stricture for hours, or perhaps even for days. South agrees with this latter view, and adds, " But I think it impossible to doubt that an additional cause is to be found in the unwarrantable, violent, and repeated squeezing which the rupture suffers during the use of the *taxis ;* so that one is only astonished that the gut is so rarely burst, and the patient destroyed in a few hours." The possibly gangrenous condition of the intestine or omentum is the great objection to this operation, and its recommendation must much depend upon the belief whether such a state of the contents of the sac is always capable of detection. Mr. Key is of opinion that parts approaching to the state of gangrene would be frequently restored if returned without the exposure to the air, &c., incidental to opening the sac. Moveover, he believes that gangrene may almost always be detected when it exists. South is of a different opinion.

" I cannot agree with Key, 'that the ordinary characters of a completely sphacelated portion of bowel are distinct enough,' for I am quite sure I have seen them all existing more than once or twice without any gangrene, but simply depending upon the unwarrantable violence used in attempting to return the rupture. But I do agree with him, that 'it sometimes happens that no such change takes place in the swelling, and then the evidence of gangrene is much more equivocal.' It is by no means infrequent to find an intestine mortified, although the time it has been strangulated is short, and not the slightest external sign leads to the presumption of its condition ; as, on the contrary, it now and then happens that the exterior of the swelling is tender, inflamed, doughy, and crackling, from the causes I have just mentioned, and yet the intestine within be healthy, and the patient recover the operation. As regards the loss of elasticity in the swelling, I believe it a very uncertain sign ; the intestine may be gangrenous, but the sac full of fluid, as is commonly the case under such circumstances, and then the elasticity remains. The only sign which I think can be relied upon, though even that is doubtful, is when the intestine has burst ; then, indeed, although the redness, doughiness, and crackling still remain, the rounding of the swelling subsides, and when a little pressure is made on it a central hollow is produced, and a sense of yielding beneath, very different from the pitting caused by pressure on œdematous cellular tissue.

" From my own personal experience of the division of the stricture external to the sac, I can say nothing, never having performed it. But I do not think so great advantage is gained by not opening the sac as is stated. From all the cases I have observed, either in the practice of others or in my own, I do not think cutting through the hernial sac, and consequently opening the peritoneal cavity, so serious as generally considered. If inflammation of the peritoneum have not been previously set up, either by the rough usage of the rupture, or by the irritation which a long strangulated or gangrenous gut produces, I cannot understand why making a small opening into the peritoneal cavity should be more dreaded than the long slits, which are now made without compunction, for the removal of diseased ovaria, and so-forth. There are, however, some conditions which even those who advise leaving the sac untouched, admit, require that it should be opened, namely, confinement of the protruded parts by entangling bands, or by adhesion to the sac itself, and a gangrenous state of the bowel. Under all circumstances, therefore, I am still disposed to continue the practice of opening the sac, as I have hitherto done, believing it to be the most safe. I cannot conclude these observations without stating that, I believe much of the fatality of operations for strangulated ruptures depends upon the *improper after-treatment.* I well recollect the time when, as soon as the patient was put to bed, he was dosed with senna and salts, with the view of speedily procuring stools, and, his already irritable bowels being thereby rendered still more irritable, he speedily sank. Although this practice is probably less followed now than formerly, yet I am afraid there is still too great inclination to employ purgatives too early. For a few hours nothing more than a clyster should be given, and not even that, unless the patient be very uneasy in his bowels, and puffed up with wind. Not unfrequently they relieve themselves, and only after 12 or 18 hours is it advisable to give medicine by the mouth, for the purpose of completely clearing the whole intestinal canal. And unless there be any special indication for calomel, I believe that castor oil is the best remedy of all." P. 47.

Mr. Teale entertains a more favorable view of the operation. After adverting to the various arguments, *pro and con,* he observes—

" My experience certainly justifies me in recommending this mode of operating wherever it is practicable, provided the necessary precautions are taken against incurring the evils to which the operation, when carelessly performed, might be exposed; and, in order to avoid these dangers, it is important to be able in the first place to recognize the symptoms which indicate the occurrence of gangrene, or of a state verging towards it; and, secondly, to guard against employing an improper degree of force for the purpose of replacing the hernia after the stricture has been divided.

" The chief indications of a gangrenous condition of the hernia are derived from the state of the tumor, and from the general condition of the patient. Discoloration of the integument, adhesion of the skin to the deep-seated envelopes, œdema or emphysema of the hernial investments, may generally be considered as indicating that the strangulated viscera are actually gangrenous, or in a state verging upon this condition. But gangrene may exist without being accompanied by these changes in the investments; when, for instance, the contact of the viscera with the sac is prevented by a copious effusion of serum into the latter, and the extension of the inflammation from the viscera to the envelopes delayed, the latter may still present a healthy aspect, even whilst the former are gangrenous; and in such cases, though the local indications of gangrene are necessarily absent, yet the constitutional symptoms will generally serve as a safe guide to the operator; the feeble pulse, the sunken and anxious countenance, and the clammy skin, rarely failing to announce the mischief."

Mr. Key lays great stress upon the transudation of a fetid odour during the operation, and forbids the external division of the sac, unless the absence of this be clearly made out, after all the other envelopes are divided. The condition bordering upon gangrene cannot be detected: but Mr. Teale observes that, we must presume its existence when intense strangulation has continued for many hours, which is most likely to occur in small herniæ. If, after exposure of the sac and division of the stricture we do not find the viscera recede easily, we must suspect their agglutination to the walls of the sac, which must therefore be opened. If, too, the parts external to the sac do not produce its constriction, this will probably be caused by the neck itself, and incision be required. To obviate the risk of returning a hernia constricted by its sac, violent pressure must be avoided, and Mr. Key recommends only such a degree as would suffice for the reduction of a non-strangulated hernia.

"In conclusion, I would again express my opinion, that the operation without opening the sac ought to be performed in all cases in which it is practicable, unless the local or general symptoms indicate the existence of gangrene or an advanced stage of inflammation. In accordance with this opinion it may be stated, as a general rule, that the operation should be attempted—1st, in most cases of large herniæ, 2nd, in many herniæ of middle size; and 3rd, in but few small herniæ, unless in the earliest stage of strangulation. As it is impossible in most cases to determine with certainty the seat of stricture before operating, the practicability of the operation must generally be doubtful. But, as has already been stated, if the attempt by this operation should fail, the sac may still be opened.

"I am informed by my friend Mr. Key that the advantages of this mode of operating have been fully borne out by his practice, and that he has not met with a single case in which any inconvenience or danger arose from not opening the sac. Mr. Key observes that he meets with but few cases of strangulated hernia, requiring operation, in which it is not desirable to avoid opening the sac, even when, from some circumstance, it is not practicable. Mr. Liston, in an obliging communication with which he has lately favoured me, states that, for several years, he has been in the habit of *trying*, in all cases of *recently* strangulated hernia, when the operation was required, to divide the stricture and return the protruded parts without opening the peritoneal sac. The risk to the patient he considers to be thereby greatly diminished." P. 118.

Mr. Teale gives an analytical table of the 32 cases in which this operation has been performed. Of these, two occurred to himself, five to Mr. Liston, six to Mr. Key, four to Mr. B. Cooper, and the remainder to different practitioners. Twenty-seven patients recovered, four died, and the fate of the other is not stated. Of these, eighteen had femoral herniæ, eleven inguinal, and three umbilical.

2. *Management of the Omentum.*—To some of the recommendations of Chelius his commentator is obliged to enter his protest. Speaking of adhesions of the omentum to the sac, M. C. says, if these occupy a considerable extent, "it must only be divided at the neck of the sac, *surrounded with some linen overspread with cerate*, and when the inflammation has subsided, it should be divided near the abdominal ring." Where there are adhesions between the omentum and the intestine, after the stricture is relieved, "the intestine must be left quiet in its place, *covered with compresses moistened in, and often wetted with, decoction of marsh-mallows*." Mr. South observes :—

"If the adhesions between the omentum and sac be old and membranous, and easily divided, it is advisable to do so, and to return the omentum. But more frequently the adhesions are too short to admit of this : or the surface of the omentum is actually glued to, or so consolidated with, the surface of the sac, that it cannot be set free without cutting through. I have had two cases of this kind, in which, having returned the gut, after freely dividing the stricture, I have left the adhering omentum undisturbed, and no ill consequences have ensued. But there is a preparation in St. Thomas' Museum, where this practice was pursued, and the gut, after division of the stricture, returned into the belly, yet the symptoms of strangulation continued, and the patient died; and on examination it was found that the omentum formed a tight cord upon the intestine as it lay transversely behind it, on the brim of the pelvis, and completely prevented the passage of the contents of the bowel through it. I do not, therefore, feel certain as to which is the best practice in such cases; but I may state, that my cases which were successful happened after (We cannot see what that has to do with the matter in question) the fatal case just mentioned. I certainly should not be disposed to adopt or recommend the practice proposed by Chelius of separating the adhesions of the neck of the sac, and passing a piece of linen round the omentum, with the purpose of dividing it at a future time, as I should expect that the presence of such extraneous substance would be likely to excite dangerous inflammation." P. 40.

In examining the omentum we must always be careful to ascertain that a small knuckle of intestine is not confined within its folds. Mr. Smith, Mr. Teale's colleague, lately met with such a case. The omentum was found continuously adherent to the whole circumference of the neck of the sac, and coiled into a long tube, the upper extremity of which embraced a small knuckle of intestine. A *small portion* of healthy omentum may be returned, but if there is a *large portion* protruded, although quite healthy, Mr. Teale agrees with most surgeons in advocating its removal. Its exposure to the air and the manipulations which would be requisite, would often induce inflammation and gangrene after its return. If contused, lacerated, gangrenous, inflamed, or anyway altered in structure it must also be removed. We must not act upon a belief in its vitality as we do in that of intestine, from the veins refilling after being emptied by pressure; for the inflamed intestine possesses a power of recovering itself when released from stricture which this part does not. In all cases Mr. Teale advocates its removal by *incision*. A temporary ligature is to be passed through it to prevent sudden retraction and consequent hæmorrhage. The portion to be removed is to be cut off by a knife or scissors, and any bleeding vessel secured by a ligature, one end being cut off close and the other allowed to pass out of the wound. After division the remaining portion is to be pushed into the abdomen, and not allowed to remain as a plug for the mouth of the sac, being when adherent there, ineffectual for the prevention of future hernia and often the cause of distressing dragging sensations at the stomach. The allowing the omentum to slough off, often gives rise to great constitutional irritation, as does its removal by the ligature alone, while the hæmorrhage resulting from simple excision renders it a dangerous practice.

"The mode of treatment which combines such a degree of efficiency and safety as to render it worthy of general adoption, is the excision of the omentum and ligature of its vessels. In reference to this practice Sir A. Cooper states, that, as far as he has seen, 'it is unattended with danger.' Mr. Lawrence speaks with the same confident tone of its safety. My own experience is in perfect accordance with theirs. Indeed, those cases in which I have either practised, or witnessed the excision of large portions of omentum, the vessels having been secured by ligature, have been among the most fortunate of the operations for strangulated hernia which have come under my notice; for the omentum had, to a certain degree, protected the intestine from the pressure of the stricture, and the removal of the omentum had not in any case been followed by evil consequences."—*Teale*, 142.

Chelius, speaking of omentum altered in structure, observes. " The general advice, in these cases, is to tie the omentum above the degenerated part, to cut it off below the ligature, to return the tied part into the belly, and to fasten the threads externally. The ligature of the omentum, however, causes a new strangulation." Mr. South states that he has tied the omentum and cut off the part below the ligature several times without any such, or other evil result following. He also states that, by tearing through as much of the omentum as he can, and dividing only that which could not be so removed, he has very rarely had occasion to apply ligatures.

" If the blood have not coagulated in the vessels of the omentum, cutting it off and tying them singly is not only an almost interminable business, but also apparently when all the vessels have been secured, and the patient put to bed, after a few hours secondary bleeding occurs from some little vessel or vessels which had escaped notice, the sac and yielding skin becoming largely distended with blood, in such quantity as to produce faintness, and require the re-opening of the wound to remove the blood and tie the bleeding vessels. This disturbance of the wound prevents the adhesive process, and very commonly gives rise to abscess of the sac or its immediate neighbourhood, by which the cure is much retarded. A case of this kind occurred to me, and a large abscess was the result, although the patient ultimately recovered. It is on this account I prefer tearing through the omentum as much as possible, by which the ends of the vessels are ensheathed in cellular tissue, and do not bleed, or even tying up the omentum together." P. 42.

3. *Trusses.*—Mr. Teale has several useful observations upon these. The use of hard pads of *wood* or *ivory*, employed in the 16th century, has been revived by the American surgeons. Dr. Chase has especially paid much attention to their due adaptation as trusses, and the Philadelphian Committee speaks highly in favour of his success. The object is not to induce inflammation and consequent consolidation of the compressed parts, but to secure the perfect retention of the viscera, thus effectually palliating the evils resulting from the disease, and enabling Nature in many instances to effect a permanent contraction of the aperture. So great is the comfort obtained by these trusses, that the Committee states, some patients refuse to discontinue them even when pronounced cured. " Pads of ivory, confined by plasters or a soft bandage, are of great value in the treatment of the *umbilical herniæ of infants.* I have also found ivory pads with a delicately adjusted spring efficient and cleanly, and not productive of pain, in the treatment of the inguinal hernia of infants. M. Malgaigne has found an ivory pad, of a mushroom form, supported by a common spring truss, of great value in some obstinate direct inguinal and umbilical herniæ." Great care must be taken that the pressure of the spring be not too forcible upon a hard pad, and that the surface of this be smooth and well adapted. Pads formed of *caoutchouc* and filled with air have been found by MM. Cresson and Sanson of great utility in supporting herniæ, manageable by no other, or where the pressure of hard bodies could not be borne. Unfortunately, they soon become useless from the escape of the air. "Caoutchouc filled with curled hair, or a solid mass of this substance, is a good substitute for M. Cresson's pads. Whenever caoutchouc is used as a compress, a layer of linen should be placed between it and the skin." Trusses constructed upon Salmon and Ody's principle have been pronounced theoretically bad, but Mr. Teale has known them most beneficially resorted to after the common spring truss has failed in giving any relief. Sir A. Cooper thought them not adapted for persons engaged in laborious employments, but M. Malgaigne states that they form most efficient instruments for this class of persons.

"It is of the greatest importance that patients should not only be furnished with an efficient truss, but also that they should be instructed in the principles upon which it acts, and the correct mode of its application. I have frequently witnessed patients whose herniæ had partially descended by the side of the truss, in consequence of the imperfect application of the instrument. The truss should always be worn both day and night. By neglect of this rule the danger of strangulation is incurred. During the simple act of turning in bed, or during the more violent efforts of coughing or yawning, the unsupported hernia may descend and become strangulated. On this account, it is desirable that the patient should be provided with two trusses, that the hernia may not be left unprotected whilst the instrument requires occasional repair.

"When the viscera are perfectly retained by trusses, the neck of the sac, no longer distended, contracts, and may become ultimately closed. So, also, the aperture in the abdominal muscles and aponeuroses, through which the hernia had descended, when no longer traversed by the protruding viscera, becomes gradually diminished, to such a degree as, in some instances, to constitute a permanent barrier to the future descent of the bowel. Thus, whilst the surgeon employs trusses as the most effectual agents in the palliative treatment of hernia, he is, at the same time, adopting means of considerable power for promoting the radical cure of the disease. But, whilst the palliative and remedial value of trusses is acknowledged, it must not be concealed that their beneficial effects are not altogether unalloyed with evil. The contraction and induration of the neck of the sac, consequent upon their use, renders it less yielding, and the abdominal viscera more liable to strangulation, if at any time they should accidentally descend." P. 74.

4. *Diagnosis of Inguinal Hernia and Hydrocele.*—Mr. Teale observes that the distinguishing inguinal hernia from hydrocele of the tunica vaginalis is usually sufficiently easy. The tumour of *hydrocele* is more pyriform; it increases from below upwards; it is usually translucent and fluctuating; but *never tympanitic.* It is rare for sufficient serum to be effused into the *hernial* sac to impart the sense of fluctuation, and still rarer for it to allow of any translucency while it *is sometimes tympanitic.* Hydrocele does not vary in size from pressure and position, which hernia frequently does. Mr. Teale, however, cautions his readers against laying too much stress upon *translucency*, as a *sign of hydrocele*, and cites two cases in point: one of these occurred to Arnaud, who was consulted for a large tumour extending from the ring to the bottom of the scrotum, and the upper part of which was divided from the lower by a circular furrow. The surgeon in attendance believed the upper division to be a rupture, and the lower a hydrocele, the light of a candle being transmitted through this last. He proposed to draw off the fluid by tapping, in order that the hernia might be reduced. Arnaud, finding that the tumour was equally painful over its whole extent, and believing it to consist of intestine distended by air to such an extent as to allow of translucency, objected to this. As neither surgeon could agree with the opinion of the other, no operation whatever was performed, and the patient was allowed to die, when Arnaud's diagnosis was found to be the correct one.

"Again, a boy was brought to me at the Leed's Infirmary with a tumour of the scrotum of large size, tense, and elastic; the integuments being very thin, from the distension which it occasioned. On examination by the aid of a candle, in the presence of several pupils, it was found to be as transparent as any hydrocele which I had seen. Whilst examining it, I observed that there were two opaque lines intersecting the tumour diagonally; which, by pressure were, to a certain extent, made to alter their situation. This circumstance, together with the comparative lightness of the tumour, and slightly tympanitic condition which I detected, excited my suspicion as to the disease being a hydrocele, and justified me in treating it as a hernia. The use of the taxis shewed that the diagnosis was correct, and that the tumour was a hernia greatly distended with flatus; for, by pressure the distended bowel was with some little difficulty replaced within the abdomen. The opaque oblique lines indicated, most probably, the point of contact of distinct folds of intestine." P. 253.

*Hydrocele of the Cord* may be more easily confounded with hernia, and cases are every now and then brought to the hospitals with trusses applied. When the tumour is situated *below* the ring, we are enabled to grasp the cord as in the normal state; and when seated within the canal, "it is distinguished from hernia by its indolent character, by its unvarying size from pressure or position, and by its exhibiting such a degree of tension as a hernia only possesses in strangulation. If doubt, however, exist respecting the nature of such a tumour from the coexistence of symptoms of intestinal obstruction, it is the safest course to expose it by an incision." Scarpa and Liston have each related a case of strangulated hernia complicated with hydrocele of the cord, and requiring operation.

5. *Inguinal Hernia in Women.*—All surgeons have hitherto stated, that femoral hernia is far more frequent than inguinal in women; but M. Malgaigne (a practitioner of vast experience in this disease) maintains that, the contrary is the fact. He admits that femoral hernia is more frequently operated upon in women than inguinal, proving only that it is more liable to strangulation. He maintains that, the supposed predominance of femoral reducible hernia is attributable to faulty diagnosis. When this was carefully attended to, he invariably found the inguinal most numerous; and of 62 female cases he examined in 1835, 54 had inguinal, seven femoral, and one both inguinal and femoral hernia. Mr. Teale observes upon this statement:—"While I am willing to admit that many inguinal herniæ have, from defective diagnosis, been reported as femoral, and am willing moreover to give M. Malgaigne every credit on this subject, I am not at present prepared to acquiesce in his statements, either from my own experience, or above that, from the experience of M. Cloquet, who examined *post-mortem* 121 women affected with hernia, and found that 42 were affected with the inguinal and 79 with the femoral varieties."

The following is the mode of examination practised by M. Malgaigne in difficult cases.

"'Reduce the hernia, feel with the right fore-finger the pulsations of the femoral artery, and, applying the pulp of the finger on the pubis side of the artery, press backwards towards the pubes. Sometimes, in thin persons, you will feel the femoral ring open, bounded in front by Poupart's ligament, behind by the pubes, on the iliac side by the vein and artery, the pulsations of the latter being felt through the interposed coats of the vein on the side of the finger; then it is unnecessary to proceed farther; in the natural state, the finger never could thus penetrate into the femoral ring. But suppose the subject to be fat, the hernia small, and the ring too deep and narrow to admit the finger; you must press against the pubes, whilst you percieve the pulsations of the artery against the side of the finger, and cause the patient to cough. If the impulse is felt by the finger, and the hernia does not escape, it is femoral; but if the impulse is not felt, and the hernia escapes above, it is inguinal. Occasionally an inguinal hernia escapes above, and at the same time communicates an impulse to the finger. This effect can only result from one of the two following causes: either it is an inguinal hernia which distends the inguinal canal, and transmits an impulse below Poupart's ligament; or the hernia is femoral, and has distended and pushed forward Poupart's ligament, and a portion of the aponeurosis of the external oblique, so as to cause a projection above the ring which you have obstructed. You then with the right fore-finger close the femoral ring, and having placed the left thumb transversely about three lines above it, cause the patient to cough, whilst you slowly withdraw the fore-finger. If the hernia be inguinal, it is thereby retained; if femoral, it protrudes.'

"These directions are only applicable to reducible herniæ. When from any cause the protrusion is irreducible, the surgeon must endeavour to trace the neck of the sac issuing from beneath Poupart's ligament. This is most effectually done (when the line of Poupart's ligament cannot be satisfactorily traced passing over the tumour) by pushing the hernia upwards, and pressing with the pulp of the finger in the direction of the femoral aperture. If the hernia be femoral, more especially if it be strangulated, a firm, resisting, and sometimes painful, substance will be found occupying the femoral ring, and preventing Poupart's ligament being felt."—*Teale*, p. 322.

We must not forget to state that Mr. Teale's work is abundantly illustrated. His plan of placing all the references at the end of the work instead of at the foot of the respective pages may be a convenient one for the printer, but is a very annoying and troublesome one for the reader.

